# Being Praised for Prosocial Behaviors Longitudinally Reduces Depressive Symptoms in Early Adolescents: A Population-Based Cohort Study

**DOI:** 10.3389/fpsyt.2022.865907

**Published:** 2022-05-17

**Authors:** Daiki Nagaoka, Nanami Tomoshige, Shuntaro Ando, Masaya Morita, Tomoki Kiyono, Sho Kanata, Shinya Fujikawa, Kaori Endo, Syudo Yamasaki, Masato Fukuda, Atsushi Nishida, Mariko Hiraiwa-Hasegawa, Kiyoto Kasai

**Affiliations:** ^1^The Department of Neuropsychiatry, The University of Tokyo, Tokyo, Japan; ^2^Research Center for Social Science & Medicine, Tokyo Metropolitan Institute of Medical Science, Tokyo, Japan; ^3^Department of Psychiatry, Teikyo University School of Medicine, Tokyo, Japan; ^4^Department of Psychiatry and Neuroscience, Gunma University Graduate School, Maebashi-shi, Japan; ^5^School of Advanced Sciences, SOKENDAI (The Graduate University for Advanced Studies), Hayama, Japan; ^6^The International Research Center for Neurointelligence (WPI-IRCN) at the University of Tokyo Institutes for Advanced Study (UTIAS), Tokyo, Japan

**Keywords:** adolescents, depressive symptoms, prosocial behavior, cohort study, longitudinal study, praise

## Abstract

**Background:**

Depression is highly prevalent and causes a heavy burden in adolescent life. Being praised for prosocial behavior might be a preventive factor because both being praised and prosocial behavior are protective against depression. Here, we investigated the longitudinal relationship between being praised for prosocial behavior and depressive symptoms in adolescents.

**Methods:**

In Tokyo Teen Cohort study (TTC), an ongoing prospective population-based cohort study, we collected 3,171 adolescents' data on self-reported experiences of being praised for prosocial behavior, depressive symptoms, and caregiver-evaluated prosocial behavior. Ten-year-old children were asked to freely describe answers to the question “What are you praised for?”. Only children who clearly answered that they were praised for their prosocial behavior were designated the “prosocial praise group.” The degree of depression at ages 10 and 12 was measured with the Short Mood and Feelings Questionnaire (SMFQ), a self-report questionnaire about depression. Objective prosocial behavior of the 10 year-old children was assessed by the Strength and Difficulty Questionnaire (SDQ). Multiple linear regression analysis was performed using the SMFQ score at age 12 as the objective variable and being praised for prosocial behavior as the main explanatory variable, and the SMFQ score at age 10 and the objective prosocial behavior at age 10 were included as confounders.

**Results:**

Depressive symptoms (SMFQ scores) in the “prosocial praise group” were significantly lower than those in the other group both at age 10 (4.3 ± 4.4 vs. 4.9 ± 4.6, *p* < 0.001) and at age 12 (3.4 ± 4.2 vs. 4.0 ± 4.6, *p* < 0.01). In the single regression analysis, the children who reported being praised for prosocial behavior at age 10 had significantly lower depressive symptoms at age 12 (partial regression variable: −0.57, 95% confidence interval (CI) [−0.96, −0.17]). This association remained significant after adjusting for confounders, including baseline depressive symptoms (partial regression variable: −0.44, 95% CI [−0.80, −0.08]). Prosocial behavior alone was not associated with depressive symptoms.

**Conclusions:**

Being praised for prosocial behavior rather than objective prosocial behavior at 10 years of age predicted lower depressive symptoms 2 years later. Praise for adolescents' prosocial behavior can be encouraged to prevent depression.

## Introduction

Depression is highly prevalent and causes a heavy burden in adolescent life (1); depression is the 8th cause of global years lived with disability (YLDs) in 10–14 years of age, 2nd in 15–19 years of age and 1st in 20–24 years of age. Thus, prevention strategies are required at all levels, including the individual, family, school, and society levels. Since praise influences self-esteem (2, 3), which is associated with depression (4), praising could prevent depressive symptoms in adolescents. An empirical research showed that parental verbal affection associated with well-being in late adolescence (5). Further, perceived praise from parents was associated with lower levels of depression in adolescents (6).

The manner and content of praise influences the self-esteem and positive attitude of adolescents. Overly positive and inflated praise has been suggested to lower self-esteem in children (3). It was also suggested that person-focused but not process-focused praise leads children to avoid challenges in school (7). Furthermore, praise for behavior (process) was better than praise for personal qualities in terms of the effect on the self-esteem of children (2). Taken together, praise for behavior may be important in preventing depressive symptoms in adolescents, but it is still unknown which behaviors of children should be praised.

A negative correlation between prosocial behavior and depressive symptoms has been noted (8). Further, since prosocial behavior predicted future decreased depressive symptoms in adolescents (9), praise for prosocial behavior is a candidate preventive strategy against depressive symptoms in adolescents. However, no study has investigated the association between recognition of being praised for prosocial behavior and depressive symptoms in adolescents. Furthermore, no study has examined whether being praised for prosocial behavior has a preventive effect on depressive symptoms in adolescents. We hypothesized that being praised for prosocial behavior would be longitudinally associated with decreased depressive symptoms in adolescents. This study aimed to examine the longitudinal relationship between being praised for prosocial behaviors and depressive symptoms using a large-scale cohort of adolescents sampled from the general population.

## Methods

### Study Design and Survey Participants

The purpose of this study was to test the hypothesis that being praised for prosocial behavior would be longitudinally associated with decreased depressive symptoms in adolescents. We adopted 10–24 years as the definition of the age of adolescence according to the recent review (10), thus called the study participants as early adolescents. This study used data from the Tokyo Teen Cohort study (TTC), a prospective population-based cohort study that is currently underway and aims to investigate the developmental trajectory of adolescents (11). Children born between September 2002 and August 2004 in three local governments in Tokyo (Setagaya, Mitaka, and Chofu) were randomly extracted using the Basic Resident Register. Of the 10,234 pairs of children and their primary caregivers who were asked to participate, 4,478 pairs agreed to cooperate in the baseline survey (the 1st wave of data collection) at the age of 10 years. An oversampling method was used with the goal of having 3,000 pairs remain in the 2nd wave survey (11). Given the low follow-up rate of low-income families, all 620 pairs whose annual household incomes was lower than five million yen were asked to participate in the 2nd wave survey. Of the remaining 3,858 pairs, 2,551 were randomly asked to participate in the 2nd wave. A total of 3,172 pairs were asked to participate in the longitudinal cohort study, resulting in 3,007 pairs participating in the second wave of data collection at the age of 12 years (follow-up rate was 94.8%).

In each of the 1st and 2nd waves of data collection, trained investigators visited the participants' home twice and interviewed children and their primary caregivers. At the first visit, the investigators explained the research to both the child and the primary caregiver, obtained written consent, and asked them to complete the self-report questionnaire by the second visit. At the second visit, the child and the primary caregiver separately answered a self-report questionnaire containing sensitive content and sealed it immediately after completion. All questionnaires and data were collected anonymously. At this visit, the investigators also administered face-to-face psychological tests to the child.

TTC is a joint study of three institutions (Tokyo Metropolitan Institute of Medical Science, the University of Tokyo, and the Graduate University for Advanced Studies) and has been approved by the ethics committees of the three institutions.

### Measures

The children answered self-report questionnaires including items on experiences of being praised, depressive symptoms and other variables, such as the number of siblings. Caregivers answered self-report questionnaires that included questions about the caregiver's age, psychological distress, educational background and annual household income.

### Experience of Being Praised for Prosocial Behavior

In the self-report questionnaire at the 1st wave, 10 year-old children were asked to freely describe answers to the question “What are you praised for?”. We dichotomized the answers to “prosocial praise group” and “other praise group.” We did not score the degree of prosociality but just divided the answers to the two groups. A qualitative classification was made by several researchers (DN, NT, HN, MT) as to whether prosocial behavior was included in the answers, double-checked by other researcher (DN, NT), and was finally confirmed by several experienced researchers (MM, TK). Based on a previous study (12), we defined prosocial behavior as voluntary, intentional behavior that results in benefits for another; the motive is unspecified and may be positive, negative, or both. Only children who clearly answered that they were praised for their prosocial behavior such as “helping with housework” were designated the “prosocial praise group,” and other children who did answer but did not include prosocial behavior in their responses such as “getting a good score on the exam” were designated the “other praise group.” Children who made multiple responses were also classified as the “prosocial praise group” if more than one of their answers included prosocial behaviors. Blank fields were treated as missing values.

### Objective Prosocial Behavior

There may be children who behave prosocially but not be praised for their prosocial behaviors. There may also be children who are praised for their prosocial behaviors but do not recognize they are praised for their prosocial behaviors. The children's recognition of being praised for prosocial behavior does not necessarily correspond to the children's objective prosocial behavior. Objective prosocial behavior of the 10 year-old children was assessed by the Strength and Difficulty Questionnaire (SDQ), for which the primary caregivers answered in the self-report questionnaire in the 1st wave of data collection. The subscale score for prosocial behavior in the SDQ was calculated (13).

### Depressive Symptoms

The Short Mood and Feelings Questionnaire (SMFQ), a self-report questionnaire about depression (14, 15), was used to investigate the degree of depression in children in the 1st (10 years old) and 2nd (12 years old) waves of data collection. Each of the 13 items had three response choices: “True” (2 points), “Sometimes true” (1 point), and “Not true” (0 points). The scores for each item were summed into the total score (0–26 points), and higher total scores meant stronger depression.

### Confounding Variables

Previous studies on the relationship between praise for children and prosocial behavior adjusted for the children's sex, age, number of siblings, caregivers' age and educational history (16–18), and one study suggested socioeconomic status as a potential confounder in future studies (18). Therefore, we included children's sex, age, number of siblings, caregivers' age and education, and annual household income as potential candidates for confounders. In addition, using external knowledge, we added children's estimated intelligence quotient (IQ) and psychological distress of primary caregivers (mainly mothers) in the 1st wave as potential candidates for confounders. Children's IQ was estimated from two subsets (Information and Picture Completion) of the Wechsler Intelligence Scale for Children (WISC-III) (19). Psychological distress of primary caregivers was assessed by the Kessler Psychological Distress Scale (K6) (20). Among these potential confounders, we regarded the variables that showed a significant association with the prosocial self-report as confounders.

### Statistical Analysis

For comparison of the demographic characteristics between the “prosocial praise group” and the “other praise group,” *t*-tests, tests of differences in population ratios, or χ^2^ tests were used. To investigate the relationship between being praised for prosocial behavior and depressive symptoms, linear regression analysis was performed using the SMFQ at the 2nd wave (12 years old) as the objective variable and being praised for prosocial behavior as the main explanatory variable. The variables which showed significant difference between the “prosocial praise group” and “other praise group” were treated as confounders. In addition, the SMFQ score at the 1st wave (10 years old) and other confounders were treated as covariates, and multiple regression analysis was performed after supplementing missing values using the multiple substitution method (number of multiple imputations: *m* = 200). Furthermore, since there is a gender difference in the development of prosocial behaviors in adolescence (18), we examined the interaction effect of sex and prosocial self-reports on depressive symptoms. An interaction term of sex X prosocial self-report was added in the multiple regression analysis. For statistical analyses, open-source statistical software R (version 3.6.1) and the multiple substitution method calculation package mice (version 3.6.0) were used.

## Results

### Demographic Characteristics

Table 1 shows the demographic characteristics of 3,007 pairs of children and primary caregivers who participated in both the 1st (10 years old) and 2nd (12 years old) waves. Regarding the question “What are you praised for?”, 845 (28.1%) children answered that they were praised for prosocial behavior (prosocial praise group), while 2,118 (70.4%) did not report any prosocial behaviors (other praise group). The prosocial praise group had a significantly higher percentage of girls (female: 56.4% vs. 43.7%, *p* < 0.001) and had more siblings (1.2 ± 0.8 vs. 1.1 ± 0.8, *p* < 0.05) than the other praise group. Depressive symptoms (SMFQ scores) in the prosocial praise group were significantly lower than those in the other praise group both at the 1st wave (4.3 ± 4.4 vs. 4.9 ± 4.6, *p* < 0.001) and at the 2nd wave (3.4 ± 4.2 vs. 4.0 ± 4.6, *p* < 0.01). The rate of children with objective prosocial behavior (subscale score for SDQ) at 10 years old was significantly higher in the prosocial praise group than in the other praise group (7.1 ± 2.0 vs. 6.5 ± 2.0, *p* < 0.001). The estimated IQ at age 10 tended to be lower in the prosocial praise group (107.2 ± 13.9 vs. 108.0 ± 14.1, *p* = 0.16). There were no significant differences in the age of children, age of caregivers, psychological distress of primary caregivers (K6 scores), educational background of caregivers, or household income between the two groups.

**Table 1:** Descriptive statistics of the study participants (*n* = 3007).

				**Being praised for**	**Being praised for**	
				**prosocial behaviors**	**other behaviors**	
	**Source**	**All participants**	**Missing**	**(*n* = 845, 28.1%)**	**(*n* = 2,118, 70.4%)**	***p*-value**
		**Mean ±SD/*n* (%)**	**(*n*)**	**Mean ±SD/*n* (%)**	**Mean ±SD/*n* (%)**
**Characteristics of child**							
Age		10.2 ± 0.3	4	10.2 ± 0.3	10.2 ± 0.3	0.279	
Female sex		1,418 (47.2)	0	477 (56.4)	925 (43.7)	<0.001^***^
Estimated IQ^a^	Child	107.7 ± 14.1	3	107.2 ±13.9	108.0 ± 14.1	0.157
Depressive symptoms^b^ at age 10	Child	4.7 ±4.6	45	4.3± 4.4	4.9 ± 4.6	<0.001^***^
Depressive symptoms^b^ at age 12	Child	3.8 ±4.5	490	3.4 ± 4.2	4.0 ± 4.6	0.001^**^
Prosocial behavior^c^ observed	Caregiver	6.7 ±2.0	10	7.1 ±2.0	6.5 ±2.0	<0.001^***^
**Family characteristics**							
Age of primary caregiver	Caregiver	42.1 ±4.2	4	42.0 ± 4.2	42.1 ±4.2	0.512
Age of primary caregiver's partner	Caregiver	44.1 ±5.1	144	43.9 ± 5.1	44.2 ±5.1	0.184
Number of siblings	Child	1.1 ±0.8	0	1.2 ±0.8	1.1 ± 0.8	0.019^**^
Psychological distress^d^ of primary caregiver	Caregiver	8.9 ±3.3	16	9.0 ±3.3	8.9 ±3.3	0.561
Educational background of primary caregiver	Caregiver						
High school or less		503 (16.7)		147 (17.4)	346 (16.3)	0.870
Vocational school or two-year college		1,314 (43.7)		362 (42.8)	936 (44.2)	
Four-year university		1,075 (35.7)		305 (36.1)	754 (35.6)	
Six-year university or graduate school		105 (3.5)		30 (3.6)	73 (3.4)	
Missing		10 (0.3)		1 (0.1)	9 (0.4)	
Educational background of primary caregiver's partner	Caregiver						
High school or less		497 (16.5)		151 (17.9)	334 (15.8)	0.624	
Vocational school or two-year college		384 (12.8)		104 (12.3)	278 (13.1)	
Four-year university		1,588 (52.8)		450 (53.3)	1,117 (52.7)	
Six-year university or graduate school		346 (11.5)		97 (11.5)	244 (11.5)	
Missing		192 (6.4)		43 (5.1)	145 (6.8)	
Annual household income	Caregiver						
0 to 2.99 million yen		130 (4.3)		33 (3.9)	96 (4.5)	0.546
3 to 4.99 million yen		452 (15.0)		134 (15.9)	304 (14.4)	
5 to 9.99 million yen		1,446 (48.1)		411 (48.6)	1,018 (48.1)	
≥10 million yen		866 (28.8)		234 (27.7)	623 (29.4)	
Missing		113 (3.8)		33 (3.9)	77 (3.6)	

*SD, standard deviation; IQ, intelligence quotient*.

*** *p < 0.001*,

** *p < 0.01 (p-value for t-test or χ^2^ test)*.

a *IQ was estimated from the two kinds of scores in the Wechsler Intelligence Scale for Children (WISC-III)*.

b *Depressive symptoms were self-reported with the Short Mood and Feelings Questionnaire (SMFQ)*.

c *Prosocial behaviors were parent-evaluated with a subscale from the Strength and Difficulties Questionnaire (SDQ)*.

d *Psychological distress was self-reported with the Kessler Psychological Distress Scale (K6)*.

### The Association Between Prosocial Self-Reports at 10 Years of Age and Depressive Symptoms at 12 Years of Age

Table 2 shows the results of simple linear regression analysis and multiple linear regression analyses to investigate the longitudinal relationship between prosocial self-reports and depressive symptoms in children. In the single regression analysis, the children who reported being praised for prosocial behavior at age 10 had significantly lower depressive symptoms at age 12 [partial regression variable: −0.57, 95% confidence interval (CI): −0.96 to −0.17, *p* < 0.01]. In the multiple regression analysis, in addition to the child's age and sex, objective prosocial behavior, the estimated IQ and the number of siblings were added as confounders because they were significantly different between the prosocial praise group and the other praise group (Table 1). Caregivers' age and education, annual household income, and psychological distress of primary caregiver (K6 scores) were excluded from confounders because there were no significant differences between the prosocial praise and other praise groups. Statistical analysis was performed after missing values were complemented by multiple substitution. Even after adjusting for confounders, being praised for prosocial behaviors at 10 years of age was significantly associated with lower depressive symptoms at 12 years of age (partial regression variable: −0.44, 95% CI: −0.80 to −0.08, *p* < 0.05). There was no evidence for the interaction effect of sex and prosocial self-report on depressive symptoms (*p* = 0.22).

**Table 2:** The association between being praised for prosocial behaviors at age 10 and depressive symptoms at age 12.

	**Unadjusted**			**Adjusted**		
	**B**	**95% CI**	***p*-value**	**B**	**95% CI**	***p*-value**
Being praised for prosocial behavior	−0.57	(−0.96 to −0.17)	0.005^**^	−0.44	(−0.80 to −0.08)	0.017^*^
Depressive symptoms at baseline^a^				0.43	(0.39 to 0.47)	<0.001^***^
Prosocial behavior at baseline^b^				−0.05	(−0.13 to 0.03)	0.221
Female sex				0.47	(0.11 to 0.77)	0.004^**^
Age in month				0.03	(−0.02 to 0.07)	0.321
Estimated IQ^c^				0.00	(−0.01 to 0.02)	0.497
Number of siblings				0.17	(−0.02 to 0.39)	0.082

*B, regression coefficient; CI, confidence interval; IQ, intelligence quotient*.

*** *p < 0.001*,

** *p < 0.01*,

* *p < 0.05*.

*Unadjusted: simple regression analysis*.

*Adjusted: multiple regression analysis (multiple assignment methods, number of multiple imputations = 200) adjusted for depressive symptoms at age 10, parent-evaluated prosocial behavior at age 10, sex, age in months at age 10, estimated IQ at age 10, and number of siblings at age 10*.

a *Depressive symptoms were self-reported with the Short Mood and Feelings Questionnaire (SMFQ)*.

b *Prosocial behaviors were parent-evaluated with a subscale from the Strength and Difficulties Questionnaire (SDQ)*.

c *IQ was estimated from the two kinds of scores in the Wechsler Intelligence Scale for Children (WISC-III)*.

## Discussion

This is the first study that investigated the longitudinal relationship between the perceived experience of being praised for prosocial behaviors and depressive symptoms using a large-scale cohort of early adolescents from the general population. Based on the multiple linear regression analyses, the self-report of being praised for prosocial behaviors at 10 years old, but not objective prosocial behavior, predicted lower depressive symptoms 2 years later even after adjusting for baseline depressive symptoms.

Self-report of being praised for prosocial behavior predicted lower depressive symptoms 2 years later in adolescents. Being praised for prosocial behavior means being praised for behavior (process) and being praised for altruism, both of which can be preventive against depression. According to the theory about praise, process praise maintains self-esteem (2). Additionally, prosociality can be preventive against depression. A previous study suggested that helping others may increase self-acceptance and self-confidence and consequently improve depression (21).

Furthermore, rather than objective prosocial behavior alone, being praised for prosocial behavior decreased future depressive symptoms in adolescents. There may be several explanations for the findings of the present study. First, being praised might be an important process of fostering self-acceptance and self-confidence as a result of prosocial behaviors. Being praised might strengthen the recognition of adolescents' prosocial behaviors, which then leads to self-acceptance. Second, being praised might prompt further prosocial behavior and attitudes, which then leads to self-acceptance. Third, this study results may reflect the relatively strong relationship between interdependent self-construal and depressive symptoms in the Japanese culture (22). Being praised for prosocial behavior might affect self-construal stronger than the prosocial behavior itself, then lead to decrease of depressive symptoms. In any case, the present study further added that the recognition of being praised for prosocial behavior might decrease depressive symptoms more than the prosocial behavior itself.

This result would not necessarily be contrary to a previous study which showed that prosocial behavior served as a protective factor against depressive symptoms in adolescents (9) because the previous study did not assess being praised for prosocial behavior. It should also be noted that the previous study targeted a social minority (Latino immigrants to the United States) with a mean age of 14.5 years, so it was not entirely consistent with the population of interest in this study.

There are several strengths of this study. First, this study revealed a longitudinal relationship between recognition of being praised for prosocial behavior and depressive symptoms with the 2 year follow-up period. Second, since this study used a large-scale (*n* = 3,007) general population sample of adolescents, a certain generalizability of the results would be assured. This is significant because it provides suggestions for intervention methods for adolescents, who are at high risk of developing depression. Third, the follow-up rate was very high (94.8%). Fourth, we conducted the analyses while including several confounders, such as depressive symptoms at baseline, estimated IQ, and number of siblings.

There are several limitations to this study related to the fact that we assessed praise for prosocial behavior by asking the children. First, we cannot determine whether the children were actually praised. There may be children who did not describe the experience of being praised for prosocial behaviors in the questionnaire but actually had an experience of being praised for such behaviors. However, on the other hand, we could assess the importance of children's recognition of being praised for their prosocial behavior in preventing depression, which would conversely be a strength of this study. Second, since the participants were 10 year-old children, a recall bias should be noted for their responses. Third, we could not investigate parenting styles or parental habit to praise which could be important factors in how children experience praise. Fourth, since the study participants were sampled only in Japan, the generalizability to other countries is questionable. Similar investigation in other countries is needed in the further research.

Clinical implications can be derived from the results of this study. With regard to preventing depressive symptoms, caregivers and professionals in relation with adolescents should consider praising prosocial behaviors when they see them. In addition, we should devise ways to praise so that children receive the message that they have been praised for their prosocial behavior. In future research, the objective assessment of praise for prosocial behavior should be considered as well as subjective assessment. Additionally, an intervention study is required to examine the effect of praising children for prosocial behaviors on preventing depressive symptoms.

## Conclusions

Being praised for prosocial behavior rather than objective prosocial behavior at 10 years of age predicted lower depressive symptoms 2 years later. Further study is required to examine the effect of praising children for prosocial behaviors on preventing their depressive symptoms.

## Data Availability Statement

The raw data supporting the conclusions of this article will be made available by the authors, without undue reservation.

## Ethics Statement

The studies involving human participants were reviewed and approved by Tokyo Metropolitan Institute of Medical Science (12–35), the University of Tokyo (10057), and SOKENDAI (Graduate University for Advanced Studies) (2012002). Written informed consent to participate in this study was provided by the participants' legal guardian/next of kin.

## Author Contributions

DN, NT, SA, MM, and TK designed the work. DN and NT conducted statistical analyses and wrote the draft of the manuscript. SA, KE, SY, AN, MH-H, and KK contributed to data acquisition. All authors reviewed the draft manuscript critically and approved the final version of the manuscript.

## Funding

This study was supported by JSPS KAKENHI (Grant Numbers JP16H06395, 16H06398, 16H06399, 17H05931, 19H00972, 19H04877, 19K17055, and 20H03596), MHLW DA Program (Grant Number JPMH20DA1001), UTokyo Center for Integrative Science of Human Behavior (CiSHuB), and International Research Center for Neurointelligence (WPI-IRCN) at the University of Tokyo Institutes for Advanced Study (UTIAS).

## Conflict of Interest

The authors declare that the research was conducted in the absence of any commercial or financial relationships that could be construed as a potential conflict of interest.

## Publisher's Note

All claims expressed in this article are solely those of the authors and do not necessarily represent those of their affiliated organizations, or those of the publisher, the editors and the reviewers. Any product that may be evaluated in this article, or claim that may be made by its manufacturer, is not guaranteed or endorsed by the publisher.

## Abbreviations

YLDs: years lived with disability
TTC: Tokyo Teen Cohort study
SDQ: the Strength and Difficulty Questionnaire
SMFQ: the Short Mood and Feelings Questionnaire
IQ: intelligence quotient
WISC: the Wechsler Intelligence Scale for Children
K6: Kessler Psychological Distress Scale
SD: standard deviation
CI: confidence interval.
