# Corrigendum: Being praised for prosocial behaviors longitudinally reduces depressive symptoms in early adolescents: a population-based cohort study

**DOI:** 10.3389/fpsyt.2023.1232335

**Published:** 2023-06-22

**Authors:** Daiki Nagaoka, Nanami Tomoshige, Shuntaro Ando, Masaya Morita, Tomoki Kiyono, Sho Kanata, Shinya Fujikawa, Kaori Endo, Syudo Yamasaki, Masato Fukuda, Atsushi Nishida, Mariko Hiraiwa-Hasegawa, Kiyoto Kasai

**Affiliations:** ^1^The Department of Neuropsychiatry, The University of Tokyo, Tokyo, Japan; ^2^Research Center for Social Science & Medicine, Tokyo Metropolitan Institute of Medical Science, Tokyo, Japan; ^3^Department of Psychiatry, Teikyo University School of Medicine, Tokyo, Japan; ^4^Department of Psychiatry and Neuroscience, Gunma University Graduate School, Maebashi-shi, Japan; ^5^School of Advanced Sciences, SOKENDAI (The Graduate University for Advanced Studies), Hayama, Japan; ^6^The International Research Center for Neurointelligence (WPI-IRCN) at the University of Tokyo Institutes for Advanced Study (UTIAS), Tokyo, Japan

**Keywords:** adolescents, depressive symptoms, prosocial behavior, cohort study, longitudinal study, praise

In the published article, there was an error.

A correction has been made to [Abstract], [Results]. This sentence previously stated:

“Depressive symptoms (SMFQ scores) in the “prosocial praise group” were significantly lower than those in the other group both at age 10 (4.3 ± 4.4 vs. 4.9 ± 4.6, *p* < 0.001) and at age (3.4 ± 4.2 vs. 4.0 ± 4.6, *p* < 0.01).”

The corrected sentence appears below:

“Depressive symptoms (SMFQ scores) in the “prosocial praise group” were significantly lower than those in the other group both at age 10 (4.3 ± 4.4 vs. 4.9 ± 4.6, *p* < 0.001) and at age 12 (3.4 ± 4.2 vs. 4.0 ± 4.6, *p* < 0.01).”

The authors apologize for this error and state that this does not change the scientific conclusions of the article in any way. The original article has been updated.

